# Glycan microarray analysis of the carbohydrate-recognition specificity of native and recombinant forms of the lectin ArtinM

**DOI:** 10.1016/j.dib.2015.11.014

**Published:** 2015-11-18

**Authors:** Y. Liu, N.T. Cecílio, F.C. Carvalho, M.C. Roque-Barreira, T. Feizi

**Affiliations:** a Glycosciences Laboratory, Department of Medicine, Imperial College London, London, United Kingdom; b Departamento de Biologia Celular e Molecular e Bioagentes Patogênicos, Faculdade de Medicina de Ribeirão Preto, Universidade de São Paulo, Brazil

**Keywords:** Glycan microarray, Lectin, ArtinM, *Artocarpus heterophyllus*, Immunomodulation

## Abstract

This article contains data related to the researc.h article entitled “Yeast-derived ArtinM shares structure, carbohydrate recognition, and biological effects with native ArtinM” by Cecílio et al. (2015) [1]. ArtinM, a D-mannose-binding lectin isolated from the seeds of *Artocarpus heterophyllus,* exerts immunomodulatory and regenerative activities through its Carbohydrate Recognition Domain (CRD) (Souza et al., 2013; Mariano et al., 2014 [2], [3]). The limited availability of the native lectin (n-ArtinM) led us to characterize a recombinant form of the protein, obtained by expression in *Saccharomyces cerevisiae* (y-ArtinM). We compared the carbohydrate-binding specificities of y-ArtinM and n-ArtinM by analyzing the binding of biotinylated preparations of the two lectin forms using a neoglycolipid (NGL)-based glycan microarray. Data showed that y-ArtinM mirrored the specificity exhibited by n-ArtinM.

## **Specification Table**

**Table:** 

Subject area	Biology
More specific subject area	Glycobiology
Type of data	Graphs and table
How data was acquired	The data were generated from a NGL-based microarray system [4]. After binding analyses, the slide was scanned using ProScanArray microarray scanner (PerkinElmer) and the image files were quantified using ScanArray Express software (PerkinElmer).
Data format	A dedicated in-house-designed software suite was used for storing, retrieving and displaying carbohydrate microarray data [5], here as histogram charts (Fig. 1) and result table (Table 1).
Experimental factors	n-ArtinM and y-ArtinM forms were biotinylated and analyzed for binding using a NGL-based microarray (in-house designation ‘Array Sets 18–22bis’) containing 255 lipid-linked glycan probes (Table 1).
Experimental features	Glycan microarray analyses of an immunomodulatory lectin
Data source location	University of Sao Paulo, Brazil and Imperial College London, UK.
Data accessibility	The data are supplied with this article and will be online available at the Web Portal of Glycosciences Laboratory, Imperial College London: https://glycosciences.med.ic.ac.uk/data.html.

**Value of the data**

• The wide spectrum of glycans that constitute the glycan microarray makes this platform suitable to compare the carbohydrate-binding specificities exhibited by native and recombinant lectins.
• The data derived from the NGL-based microarray analyses provide important information on the carbohydrate binding specificities of y-ArtinM and n-ArtinM, and serve as the basis for further studies on the fine specificities of the lectins using other microarray systems or complementary techniques.

## 1. Data

**Fig. 1:**
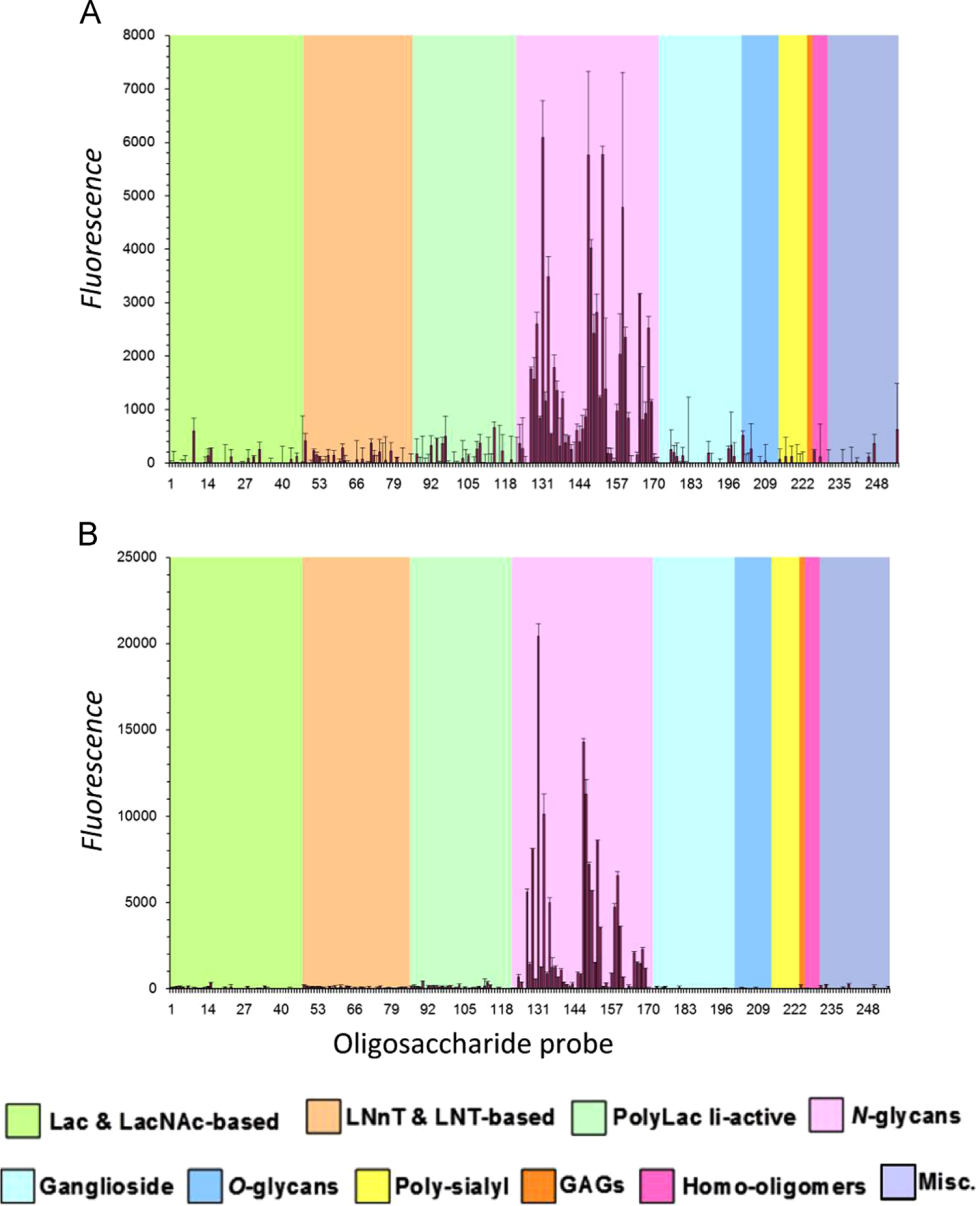
Carbohydrate microarray analyses of n-ArtinM (*A*) and y-ArtinM (*B*)**.** Numerical scores of the binding signals are means of duplicate spots at 7 fmol/spot (with *error bars*). The complete list of probes and their sequences and binding scores are in Table 1.

In this study, we analyzed the native form of ArtinM and its yeast-derived counterpart, in terms of their ability to bind to 255 glycans distributed in a microarray platform, in order to identify whether n-ArtinM and y-ArtinM shared sugar-recognition specificity. Measurement of fluorescence intensity indicated that both preparations bound to *N*-glycan-related sequences (Fig. 1A and B), with a preference for probes having the core trimannoside Manα1-3(Manα1-6) Man. This binding intensity was enhanced when the probe contained a Fucose residue at the trimannoside core (Table 1 – probe 131); whereas binding was diminished when a similar position in the glycan was occupied by β1-2-linked xylose (probes 130 and 132). Some differences between the two lectin forms were identified in the magnitude of binding to probes 129, 131, 133, 135, 147, 148, 149, 150, 152, 153, 158, 159 and 160. In general, y-ArtinM showed higher fluorescence intensity than n-ArtinM.

**Table 1:** Oligosaccharide probes included in the microarray and the binding signals (means of the fluorescence intensity at 7 fmol/probe spot) of n-ArtinM and y-ArtinM.

## 2. Materials and methods and data

### 2.1. Sample preparation

n-ArtinM was obtained from a saline extract of *Artocarpus heterophyllus* (jackfruit) seeds [6]. *Saccharomyces cerevisiae* BJ3501 was used to express y-ArtinM and the lectin was obtained by yeast lysis [1]. n-ArtinM and y-ArtinM were purified by affinity chromatography on a D-mannose column coupled to AKTA Purifier (GE Healthcare, Bio-Science Inc. Germany), previously equilibrated with phosphate-buffered saline (PBS) containing 0.5 M NaCl. After washing with equilibrating buffer, the adsorbed material was eluted with 0.1 M D-mannose in equilibrating buffer. The preparations obtained were ultradiafiltered against PBS using a YM10 membrane (Amicon Division, W.R. Grace, Beverly, MA) and biotinylated using sulfo-NHS-LC-biotin (Sigma-Aldrich, St. Louis, USA) according to the manufacturer instructions.

### 2.2. Glycan microarray analyses

Microarray analyses were performed using the neoglycolipid (NGL)-based system [4], with lipid-linked glycan probes, including NGLs and glycolipids, and comprising a total of 255 oligosaccharides (in-house designation ‘Array Sets 18–22bis’; list of probes are in Table 1). These were robotically printed on nitrocellulose-coated glass slides, at 2 and 7 fmol per spot, using a non-contact arrayer (Piezorray; PerkinElmer LAS, Beaconsfield, UK). The microarray binding assays were performed as described [1]. In brief, microarray slides were blocked at ambient temperature with 1% w/v bovine serum albumin (BSA; Sigma-Aldrich) in casein blocker solution (Pierce Chemical Co, USA) for 1 h. The biotinylated lectin samples were overlaid at 50 μg/mL, and binding was detected using Alexa Fluor 647-labeled streptavidin (Molecular Probes-Life Technologies, CA, USA) at 1 μg/mL in blocker solution. Glycoarray data analysis was performed with dedicated software [5]. The binding signals were probe-dose dependent. The results of glycan probes at 7 fmol per spot are shown in Fig. 1 and Table 1.

## Funding sources

This study was supported by Grants from the Fundação de Amparo a Pesquisa do Estado de São Paulo (2009/16146-9; 2006/60642-3, and 2013/04088-0), Conselho Nacional de Desenvolvimento Científico e Tecnológico (306503/2009-3; 306298/2013-9), Financiadora de Estudos e Projetos (0110045900), by the United Kingdom Research Council Basic Technology Initiative Glycoarrays and Translational Grants GRS/79268 and EP/G037604/1, and by Wellcome Trust Grants WT093378MA and WT099197MA (to T F).
